# Supplementary material for: Structure–Property Relationship in Melt-Spun Poly(hydroxybutyrate-co-3-hexanoate) Monofilaments
Source: Polymers (Basel). 2022 Jan 4;14(1):200. doi: 10.3390/polym14010200 (PMC8747132; doi:10.3390/polym14010200)
Supplement: Supplementary file 1 [file polymers-14-00200-s001.zip › polymers-1515308-supplementary-done.pdf]

polymers

Supporting information:

Structure-property relationship in melt-spun Poly(hydroxybutyrate-co-3-hexanoate) monofilaments

Figen Selli[1], Rudolf Hufenus[2], Ali Gooneie[2], Umit Halis Erdoğan[1], Edith Perret[2,3]

[1]Dokuz Eylul University, Department of Textile Engineering, Izmir, Turkey
[2]Laboratory for Advanced Fibers, Empa, Swiss Federal Laboratories for Materials Science and Technology, Lerchenfeldstrasse 5, 9014 St. Gallen, Switzerland
[3]Center for X-ray Analytics, Empa, Swiss Federal Laboratories for Materials Science and Technology, Überlandstrasse 129, 8600 Dübendorf, Switzerland

WAXD analysis

Figure S1 shows almost the same Figure as Figure 12 of the main article, but it also includes the values (tensile strength, peak heights of α(020), P_{nc} reflection) of fibers measured directly after spinning in faded colors.

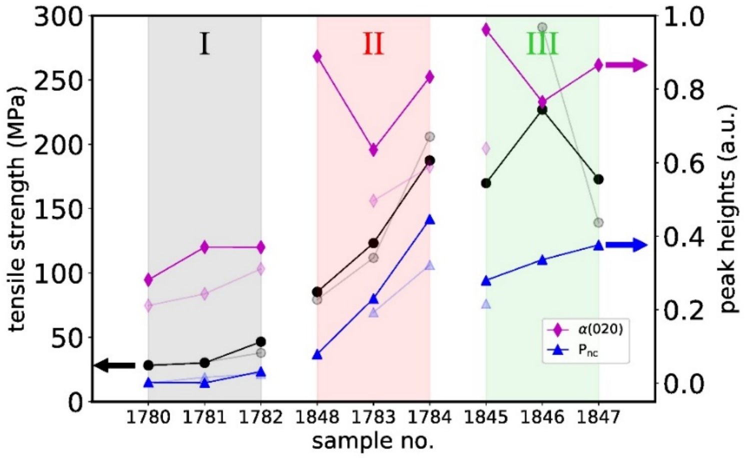

Figure S1. Tensile strength and peak heights of α(020) and P_{nc} in azimuthal profiles as a function of sample number.

Values of aged (33 months) fibers are shown in full colors and measured values directly after spinning in faded colors.

SAXS analysis

2D SAXS patterns of melt-spun PHBH monofilaments are shown in Figure S2. Two-point reflections are visible on the meridian, which points along the fiber axis (vertical).

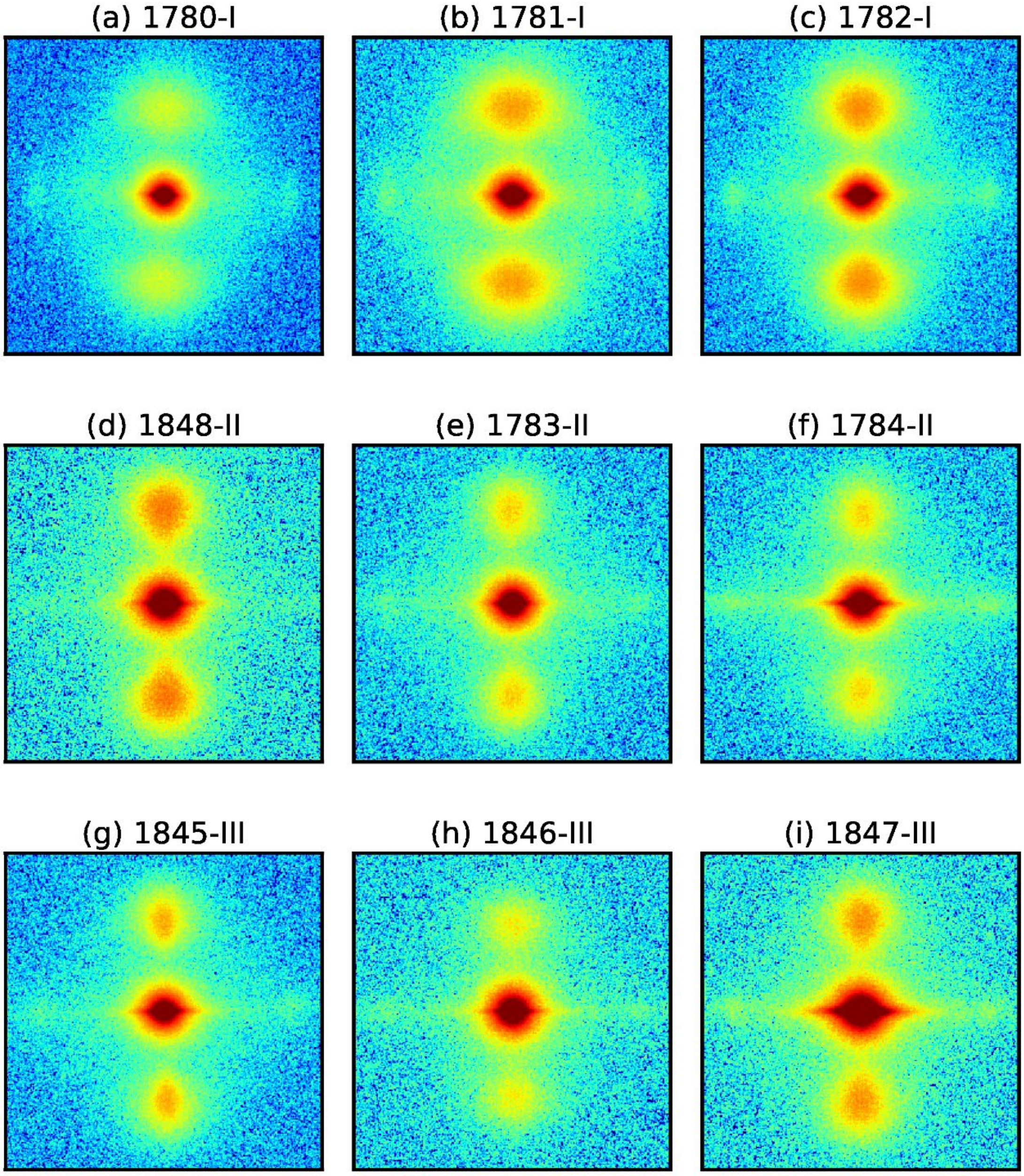

Figure S2. 2D SAXS patterns of melt-spun PHBH monofilaments for polymer grade **I** (a-c), **II** (d-f), **III** (g-i).

The meridional profiles, shown in Figure S3, have been extracted by projecting the SAXS patterns onto the meridional (vertical) axis [1]. The profiles have been fit with Pearson VII functions in order to calculate the average long-spacings from the positions of the reflections and the coherence lengths from the widths of the reflections along the meridian [2, 3]. The extracted parameters are shown in Figure S4.

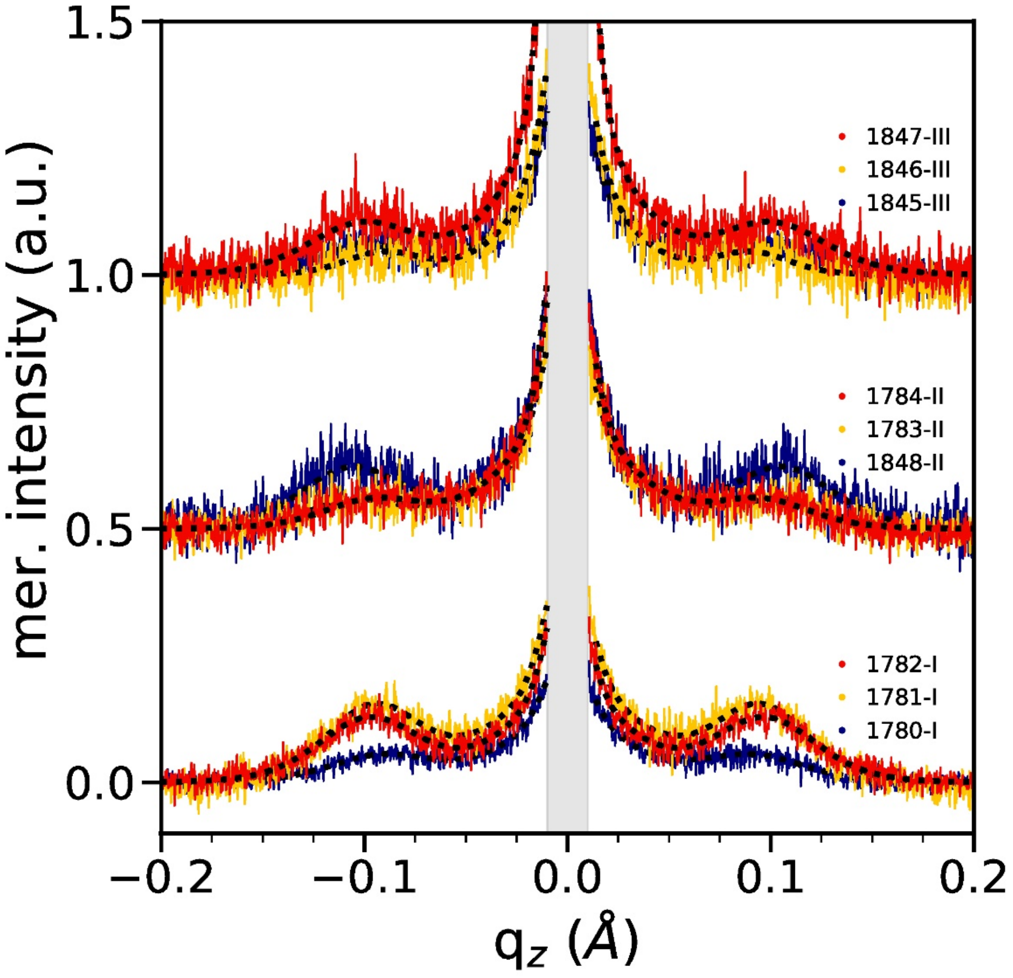

Figure S3. Meridional profiles of PHBH monofilaments. Fits are shown as black dots.

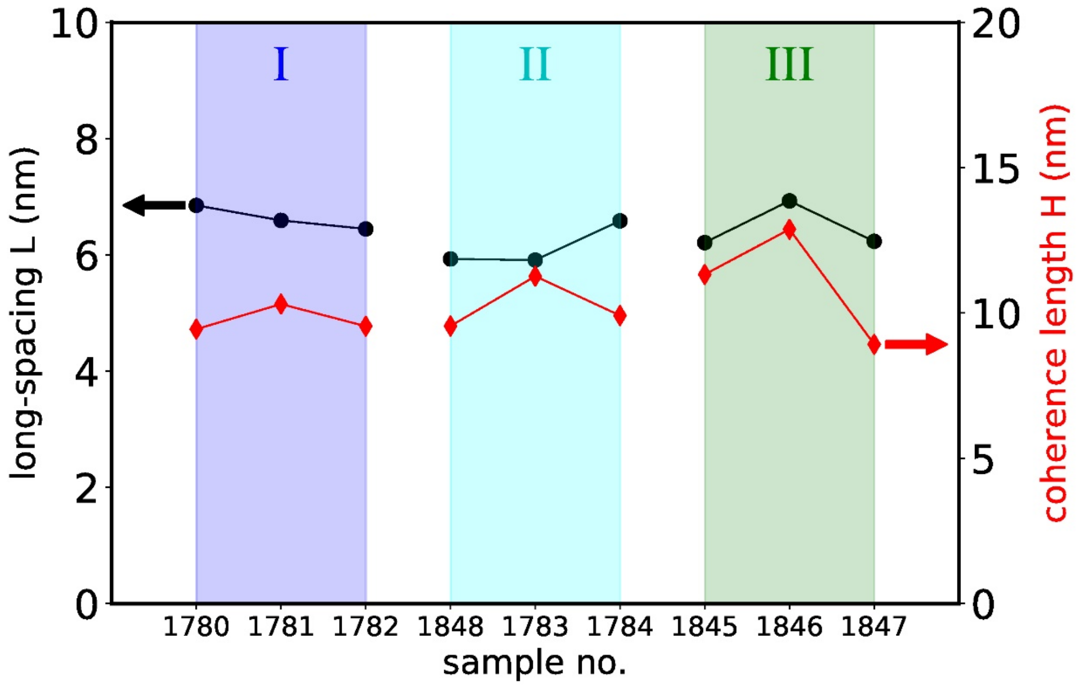

Figure S4. Long-spacings and coherence length as a function of sample number.

1. Stribeck, N., *Analysis of SAXS Fiber Patterns by Means of Projections*, in *Scattering from Polymers*. 1999, American Chemical Society. p. 41-56.
2. Perret, E., et al., *X-ray data from a cyclic tensile study of melt-spun poly(3-hydroxybutyrate) P3HB fibers: A reversible mesophase.* Data in Brief, 2019. **25**: p. 104376.
3. Perret, E., et al., *Tensile study of melt-spun poly(3-hydroxybutyrate) P3HB fibers: Reversible transformation of a highly oriented phase.* Polymer, 2019. **180**: p. 121668.
